# Supplementary material for: Funding and COVID-19 research priorities - are the research needs for Africa being met?
Source: AAS Open Res. 2020 Nov 24;3:56. [Version 1] doi: 10.12688/aasopenres.13162.1 (PMC7927206; doi:10.12688/aasopenres.13162.1)
Supplement: Peer review history

# Peer review: Reviewer response for version 1

**DOI:** 10.21956/aasopenres.14267.r28392

**Published:** 2021-04-09

**Authors:** Simon Kay

**Affiliations:** 1 International Operations and Partnerships, Wellcome Trust, London, UK

This article provides a narrative summary and description of the state of play of the funding for COVID-19 research across the African continent. It describes a living systemic review (LSR) of the research in play at the time of analysis, according to the UKCDR/GloPID-R tracker as at 15th July 2020. The analysis revealed limited COVID-19 research across Africa, with the majority of funding from Europe, surprisingly little from the US and none (as revealed by the tracker) from African sources. Whilst many projects mapped to the WHO research priorities, five gaps in research funding were identified. 

 Avoiding repeating good points made by other reviewers, for this reviewer the questions prompted by this paper are:

- Does the absence of African funding of COVID-19 research mitigate against funding of priorities relevant and important for African populations?
- I am not sure if the paper really gets at why the research needs of Africa are not being met. Is it because of quantity of funds, how funding decisions are made; or simply because it was the early days of the pandemic with external funders focused on their own needs and populations?
- Does the absence of African funding undermine African ownership of how research funding is targeted and in particular if research is conducted in very low resource settings?
- Could a future study look at how existing capacity and existing networks have been repurposed to focus on COVID-19, just strengthening the arguments for building capacity?

Is the work clearly and accurately presented and does it cite the current literature?

Yes

If applicable, is the statistical analysis and its interpretation appropriate?

I cannot comment. A qualified statistician is required.

Are all the source data underlying the results available to ensure full reproducibility?

Yes

Is the study design appropriate and is the work technically sound?

Yes

Are the conclusions drawn adequately supported by the results?

Yes

Are sufficient details of methods and analysis provided to allow replication by others?

Yes

Reviewer Expertise:

My expertise is in funding health sciences research and building health research capacity. I hold a PhD in Immunology.

I confirm that I have read this submission and believe that I have an appropriate level of expertise to confirm that it is of an acceptable scientific standard.

# Peer review: Reviewer response for version 1

**DOI:** 10.21956/aasopenres.14267.r28394

**Published:** 2021-03-02

**Authors:** Jean Marie Vianney Habarugira

**Affiliations:** 1 European and Developing Countries Clinical Trials Partnership (EDCTP), The Hague, The Netherlands; 2 Universitat Autònoma de Barcelona (UAB), Barcelona, Spain

This paper describes the COVID-19 research funding landscape in Africa as of 15 July 2020. As a baseline analysis, this is a very important reference for all stakeholders engaged in research response across Africa. By measuring investments against the research priorities outlined in the WHO Global Roadmap, the authors have opened an urgent and necessary discussion: the research needs specific to Africa, now and in the years to come.

 This baseline analysis should have an update or follow up publications to capture the very fast-growing mountain of COVID-19 research data. Recommendations to the authors and all those carrying out similar analysis, include (but are not limited to) the following: 

- Efforts should be made to map the size and type of investments made by Governments to ensure a certain level of health system's readiness to absorb research activities during an outbreak; many governments in Africa have provided cash or in-kind contributions to their outbreak response teams, and the same teams are directly or indirectly involved in research projects. This may be hard to quantify but should be captured in further analysis, where possible.
- An important role has been and is being played by various networks and consortia that started conducting epidemic preparedness research activities long before COVID-19. Consortia such as ALERRT and PANDORA-ID-NET with a strong presence in Africa, went into research response mode in the very early days of the pandemic. It would be good to measure the efficiency of these pre-COVID initiatives, how their quick research response aligned with the recommendations from the WHO Roadmap which informed further researcher strategies.
- As the vaccine roll out unveils again the inexcusable inequity across the globe, follow up analysis should provide insights into vaccine-related knowledge gaps in the African context. AAS, Africa CDC, WHO Afro and others are certainly taking quick steps in generating and disseminating information on data to be collected following introduction of vaccines, but the larger community of funders and researchers must accompany these efforts with a continued and solid funding base targeting research priorities specific to Africa.  

Is the work clearly and accurately presented and does it cite the current literature?

Yes

If applicable, is the statistical analysis and its interpretation appropriate?

Not applicable

Are all the source data underlying the results available to ensure full reproducibility?

Yes

Is the study design appropriate and is the work technically sound?

Yes

Are the conclusions drawn adequately supported by the results?

Yes

Are sufficient details of methods and analysis provided to allow replication by others?

Yes

Reviewer Expertise:

Clinical Research capacity (focus on Africa), Epidemics Preparedness, Infectious diseases, research funding strategies for LMICs

I confirm that I have read this submission and believe that I have an appropriate level of expertise to confirm that it is of an acceptable scientific standard.

# Peer review: Reviewer response for version 1

**DOI:** 10.21956/aasopenres.14267.r28227

**Published:** 2021-01-13

**Authors:** Nelson Sewankambo

**Affiliations:** 1 Lown Scholars Program, Harvard T.H. Chan School of Public Health, Boston, MA, USA

Using Microsoft Excel 2019, the authors carried out a comprehensive, descriptive and thematic analysis of the COVID-19 funded research projects in Africa initiated by COVID CIRCLE and listed in the UKCDR/GloPID-R Tracker from the start of the pandemic to 15th July 2020. The analysis revealed significantly lower funding investments in COVID-19 research in Africa compared to high-income countries, seven months into the pandemic. This finding suggested a paucity of research targeting the research priorities of relevance to Africa.

Is the work clearly and accurately presented and does it cite the current literature?

Yes

If applicable, is the statistical analysis and its interpretation appropriate?

Not applicable

Are all the source data underlying the results available to ensure full reproducibility?

Yes

Is the study design appropriate and is the work technically sound?

Yes

Are the conclusions drawn adequately supported by the results?

Yes

Are sufficient details of methods and analysis provided to allow replication by others?

Yes

Reviewer Expertise:

Building research capacity, Infectious Diseases, non communicable diseases,  and ethics

I confirm that I have read this submission and believe that I have an appropriate level of expertise to confirm that it is of an acceptable scientific standard.
